# Stability and Repeatability of the Distress Thermometer (DT) and the Edmonton Symptom Assessment System-Revised (ESAS-r) with Parents of Childhood Cancer Survivors

**DOI:** 10.1371/journal.pone.0159773

**Published:** 2016-07-25

**Authors:** Tatsiana Leclair, Anne-Sophie Carret, Yvan Samson, Serge Sultan

**Affiliations:** 1 Department of Hematology-Oncology, Centre Hospitalier Universitaire (CHU) Sainte-Justine, 3175, Chemin de la Côte Sainte-Catherine, Montréal, Québec, H3T 1C5, Canada; 2 Department of Psychology, Université de Montréal, Pavillon Marie-Victorin, 90, avenue Vincent d'Indy, Montréal, Québec, H2V 2S9, Canada; University of York, UNITED KINGDOM

## Abstract

**Objective:**

Parents report psychological distress in association with their child's cancer. Reliable tools are needed to screen parental distress over the cancer trajectory. This study aimed to estimate the stability and repeatability of the Distress Thermometer (DT) and the Depression and Anxiety items of the Edmonton Symptom Assessment System-revised (ESAS-r-D; -A) in parents of children diagnosed with cancer.

**Methods:**

Fifty parents (28 mothers, median age = 44) of clinically stable survivors of childhood solid and brain tumours completed questionnaires about their own distress (DT, ESAS-r-D; -A, Brief Symptom Inventory-18: BSI-18, Patient Health Questionnaire-9: PHQ-9, Generalized Anxiety Disorder-7: GAD-7) and their children’s quality of life (QoL; Peds Quality of Life: PedsQL) twice, with a month interval between the two assessments. At retest, parents also evaluated life events that occurred between the two time points. Hierarchical regressions explored moderators for the temporal stability of test measures.

**Results:**

Stability estimates were ICC = .78 for the DT, .55 for the ESAS-r-D, and .47 for the ESAS-r-A. Caseness agreement between test and retest was substantial for the DT, fair for the ESAS-r-D, and slight for the ESAS-r-A. Repeatability analyses indicated that the error range for the DT was more than 2 pts below/above actual measurement, whereas it was more than 3 pts for the ESAS-r-A, and 2.5 for the ESAS-r-D. Instability of the DT could be explained by changes in children’s physical QoL, but not by other components of QoL or life events. No moderators of stability could be identified for the ESAS-r items.

**Conclusions:**

The DT appears to be a fairly stable measure when the respondent's condition is stable yet with a relatively wide error range. Fluctuations in distress-related constructs may affect the temporal stability of the DT. The lower stability of ESAS-r items may result from shorter time-lapse instructions resulting in a greater sensitivity to change. Findings support future research on the DT as a reliable instrument in caregivers.

## Introduction

Caring for a child with cancer is a distressing experience, which can affect parents in the long-term. Beyond treatment termination, parents continue to be exposed to illness-related stressors such as uncertainty about cure/relapse [1], physical or emotional late effects [2], and risk of a second cancer [3]. A recent review suggested that even though most parents are resilient, a substantial subgroup of parents of survivors report clinical levels of distress, severe traumatic stress, and worries regarding their child's health beyond five years post-diagnosis [4]. Studies have described parents' difficult adjustment particularly when their child had received intense treatments, such as in the care of brain tumour patients [5, 6]. With parents being the primary caregivers of their child, it is paramount to address their needs accurately to promote family resilience. In a context where support care resources are rare, developing reliable screening tools to identify distress in parents is an important task.

Screening for distress allows professionals to better match resources to family needs thus allowing for appropriate stewardship of available resources and more effective allocation of intervention [7]. However, in spite of paediatric standards of care recommending family support, no systematic distress screening strategy is currently being implemented in paediatric oncology [8, 9]. This may potentially leave many families with untreated psychosocial difficulties. North-American governmental agencies [10, 11] have recommended using the Distress Thermometer (DT) [10] and the Edmonton Symptom Assessment System (ESAS) [12] to screen for distress in adult oncology. The DT has been used with caregivers both in adult [13, 14] and paediatric oncology [15–17]. The ESAS has been employed as a caregiver proxy measure to describe patient distress but not caregiver status. Most research thus far on the DT has addressed its validity and feasibility, with only one study dedicated to its reliability [18]. This study assessed test-retest reliability or temporal stability with cancer patients over a one-week period. A stability of *r* = .80 was reported, which was considered as an “acceptable” level. The stability of the ESAS and its revised version ESAS-r [19] has been studied in a number of reports. Stability over a one-week period was *ρ* = .54 (ESAS-D) and *ρ* = .35 (ESAS-A) [20]. Logically, coefficients were larger for a one-day interval [20–22]. However, the interpretations of these stability levels were not based on an analysis of the measured construct, participant characteristics, or time intervals, or systematic analyses of repeatability [23]. Most also used correlation as an estimate of stability whereas differences and agreement statistics are better suited for stability and repeatability estimation.

Considering the significant variability reported in the literature on parental distress with childhood cancer [24], reliable instruments are all the more so required to prevent additional instability from blurring assessments. Several factors may influence the stability of such tools, including changes in distress in relation to the child’s status, other occurring life events, or factors associated with the measures’ reliability or inherent sensitivity. Documenting reliability of tools such as the DT or ESAS is of primary importance as users are increasingly treating distress levels as outcomes or predictors of clinical change, independently from their screening abilities [25, 26]. Stability over time is the only method to ascertain reliability for such one-item tools. In a situation where change is not likely to occur, one would expect good-excellent stability levels in reliable instruments, and instability could be attributed to lack of reliability. Another interesting property is sensitivity. Sensitivity is best studied when change is expected and it can be ascertained within concurrent validity or criterion validity designs.

This study had three objectives. 1) To evaluate the temporal stability of the DT and the ESAS-r-D and -A (i.e., *test measures*) in parents of children survivors of cancer, and compare them with the stability of other existing tools (i.e., *validity measures*). 2) To explore repeatability and estimate the measurement error and error range of the test measures. 3) To explore stability moderators, by assessing the effect of changes in the child's quality of life (QoL) and life events over the time interval. We expected that the measures would show good or excellent stability (i.e. ICC > 0.60) and that the DT would show larger stability than the ESAS-r items, in line with time frames (DT: one week, ESAS-r: one day). We expected that instability (or change) in test measures would be associated with changes in children’s QoL and life events during the month's period.

## Methods

### Participants and procedure

Data were collected between July and December 2014 at the Hematology-Oncology department of a Canadian paediatric hospital (CHU Sainte-Justine, Montreal). Sixty-one cancer survivors were randomly selected from a list of 187 patients, diagnosed between 1999 and 2009 (< 18 years old, solid or brain tumour diagnosis, ≥ five years post diagnosis, in remission, see Table 1). Due to other research projects involving families of children with diagnoses of blood cancers, these families were not available for the present project and we concentrated on solid and brain tumor diagnoses. We contacted parents (i.e., any adult responsible for the child) by telephone (*N* = 80). To be eligible, parents had to have been involved in the child's treatment since diagnosis, and be able to read French or English. Sixty-five parents agreed to participate (81% acceptance rate). They were sent consent forms and questionnaires by surface mail. Non-responders were parents who did not return calls. Two parents declined participation. Fifty-six parents returned the test assessment (87% response rate). No difference was observed between responders and non responders on parental age, child age, diagnosis, or treatment type. Fifty-three parents returned the retest assessment (5% attrition). Three parents were excluded at retest due to missing data or unstable child medical condition. Consequently, analyses include 50 participants. All children were younger than 18 and the range in time off-treatment was 2–13.5 yrs. Fifteen (45%) needed a special education program). Approval was obtained by the Research Ethics Board of the CHU Sainte-Justine.

**Table 1:** Demographic Information.

Parents (*n* = 50)	*M (SD)*	*n*	*%*	Children (*n* = 33)	*M (SD)*	*n*	*%*
Mothers		28	56	Girls		12	36
Fathers		20	40	Boys		21	64
Grandparents		2	4	Age	11.70 (3.05)		
Parents in couple		34	68	5–7		2	6
Age ^a^	44.06 (5.71)			8–12		16	49
30–39		10	21	13–17		15	45
40–49		30	63	Age at diagnosis	2.79 (2.48)		
50–59		8	16	Time since diagnosis (years)	8.91 (2.44)		
Origin				Solid tumours		25	76
Canadian		45	90	Hepatoblastoma		2	6
Other		5	10	Histiocytosis		4	12
Education (obtained diploma)				Neuroblastoma		8	25
None		1	2	Retinoblastoma		1	3
Secondary		14	28	Germ cell tumour		3	9
College		14	28	Wilm's tumour		7	21
University		20	40	Brain tumours		8	24
Missing		1	2	Astrocytoma glioma		1	3
Income				Craniopharyngioma		2	6
< 20,000 $		9	18	Optic nerve glioma		1	3
20,000–40,000 $		8	16	Medulloblastoma		3	10
40,000–60,000 $		20	40	PNET^b^		1	3
60,000–80,000 $		4	8	Treatment			
> 80,000 $		7	14	Chemotherapy		24	73
Missing		2	4	Radiotherapy		15	45
				Surgery		29	88
				Relapse		5	15

^a^ excluding grand-parents (age: 59 and 63)

^b^ PNET, Primitive neuroectodermal tumour.

### Materials

Demographic questionnaire. This included parents' demographic and family information, and child medical history and current health status (S1 File). A child was clinically stable when the parent reported at least two criteria of the following: 1) no current relapse, 2) stable health status in the last month, 3) no health complications in the last month.

Distress Thermometer (DT) [10]. The DT consists of an 11-point numeral scale (0 = *No distress*; 10 = *High distress*), on which participants are asked to rate the distress they have experienced over the last week. The instrument includes a problem list of 34 problems ranging from practical, emotional, parenting, family or social, physical, or cognitive problems. This is also complemented by a brief inquiry of needs in 5 additional items. This version of the DT has been validated with parents of children receiving treatment [16]. The score was strongly associated with the total score of the Hospital Anxiety and Depression Scale, yielding an optimal cutoff of ≥ 4 [27].

Edmonton Symptom Assessment System-revised (ESAS-r) [20]. The ESAS-r includes nine items, answered on 11-point numeral scales, and an optional blank item for patient-specific symptoms (0 = *No symptom*; 10 = *Worst possible symptom*). Participants are asked to evaluate symptoms over the current day. We used the Depression and Anxiety items. These items have cutoffs of ≥ 2 and ≥ 3 with outpatients, and were strongly associated with the Patient Health Questionnaire-9 and the Generalized Anxiety Disorder-7 [13]. The ESAS has been validated in French [28].

Brief Symptom Inventory-18 (BSI-18) [29]. The BSI-18 is a screening measure of anxiety and depression symptoms. It assesses distress over the last week on 18 items on 5-point scales (0 = *Not at all*; 4 = *Extremely*). The measure includes three subscales of 6 items: Somatization (SOM), Depression (DEP), Anxiety (ANX), and a general distress score (Global Severity Index, GSI). Standard cutoffs are available to evaluate the risk of caseness. High internal consistency has been reported, which was comparable in our sample (subscales: *α* = .80-.87, GSI: .94). The GSI appeared to have moderate stability and was strongly associated with the Beck Anxiety Inventory and the Beck Depression Inventory in outpatients [30].

Patient Health Questionnaire-9 (PHQ-9) and Generalized Anxiety Disorder-7 (GAD-7) [31]. The PHQ-9 (nine items) and the GAD-7 (seven items) are screening measures that evaluate the intensity of depressive and anxious symptoms over the last two weeks on 4-point scales (0 = *Not at all*; 3 = *Nearly every day*). Scores to items are summed and totals of ≥ 10 indicate moderate symptoms. Moderate to high reliability was reported for the PHQ-9 [32] and the GAD-7 [33]. Internal consistency in our sample was also high (PHQ-9: *α* = .84, GAD-7: *α* = .86). In the general population, the PHQ-9 was strongly associated with the Brief Beck Depression Inventory [34] and the GAD-7 was moderately associated with the Rosenberg Self-Esteem Scale [35].

PedsQL 4.0 Generic Core scales: parent proxy-report (PedsQL) [36]. The PedsQL assesses the child’s quality of life over the last month. It includes 23 items, distributed on four scales: Physical (eight items), Emotional (five items), Social (five items) and School (five items), for a total of 23 items, rated on 5-point scales (0 = *Never*; 4 = *Almost always*). Internal consistency for the total score is very high in paediatric cancer [37], and similar in our sample (*α* = .92; scales: *α* = .74–89). Stability of the scales appeared high with children hospitalized for traumatic brain injury [38]. The measure distinguished children with cancer from healthy children and children on-treatment from those off-treatment children [37]. Change in child QoL was calculated as the difference between test and retest levels.

Life Experiences Survey (LES) [39]. The LES is a life events inventory that measures exposure to stress. We used the general events section of 47 items. Participants are asked to check off life events that they have experienced over the last year, and rate their impact on a 7-point scale (-3: *Extremely negative*, 3: *Extremely positive*). Negative and positive items are summed to yield a negative and a positive change score [40].

### Statistical analysis

As preliminary analyses, we conducted Receiver Operating Characteristic (ROC) curve analyses to determine optimal cutoffs for the DT and the ESAS-r-D and -A to detect distress, depression and anxiety against the BSI-18, PHQ-9, and GAD-7, respectively. We performed descriptive statistics for all measures at test and retest (*M*, *SD*). For objective 1, we used intraclass correlation coefficients (ICCs) to estimate stability and classified values as poor (ICC < .40), fair (.40-.59), good (.60-.74), and excellent (.75–1.00) [41]. We used paired samples *t*-tests and effect sizes *d* to estimate mean-level changes. To examine stability of caseness, we used cross tables with chance corrected Kappa coefficients. Caseness was defined in reference to pre-validated cutoffs and sample-specific cutoffs. For objective 2, for each test measure, we calculated the mean and standard deviation of T2-T1 differences and determined the limits of agreement (M ± 1.96*SD) which indicates the interval in which 95% of differences lie. We used the Mean to Difference plot and the Kendall’s τ to examine relationships of instability with levels on the measures. We computed the measurement error (SD/√2) and the error range (SD/√2*1.96). The error range indicates that the average of all possible measurements of the test measure is within the range of the value of the error below/above the actual measurement taken. For objective 3, we examined the role of potential moderators on instability for each test measure with hierarchical regressions. Test measures (retest) were entered as the dependent variable, test measures (test) as the first block, and life events and change in child QoL were entered as alternate second blocks. Because of skewed distributions, Life events scores were recoded according to the median for regression analyses.

A power analysis was performed to estimate the required sample size to test the value of the stability coefficient >.60, with a one-tail z-test model, an alpha level of 0.05 and power of .95, the required sample size was N = 42.

## Results

### Preliminary analyses

At baseline, 8% of parents met case criteria on the BSI-18, 12% on the PHQ-9, and 6% on the GAD-7, in comparison to 10% [29], 9% [34], and 3% [35] in the general population. Following ROC curve analyses, optimal cutoffs in our sample were: ≥ 3 for the DT when detecting distress against the BSI-18, PHQ-9, and GAD-7, ≥ 3 for the ESAS-r-D when detecting depression against the PHQ-9, and ≥ 5 for the ESAS-r-A when detecting anxiety against the GAD-7 (S1–S5 Tables). Consequently, 32%, 36%, and 18% of parents reported case levels of distress on the DT, ESAS-D and ESAS-A, respectively (Table 2). Test measures were strongly associated at both times (*r*s = .75-.88), and were also closely associated with validity measures (*r*s = .68-.83).

**Table 2:** Stability and repeatability estimates.

	T1			T2			Comparisons			
	*n*	*M* (SD)	Case *n (%)*	*n*	*M* (SD)	Case *n (%)*	*Mdiff* (SDdiff)	*d*	*ICC*	*κ*
**Test measures**^**a**^										
DT	50	1.72 (2.01)	16 (32)	48	1.90 (2.46)	13 (27)	0.15 (1.52)	0.10	0.78	0.75
ESAS-r-D	50	1.90 (1.99)	18 (36)	49	1.55 (1.87)	12 (24)	-0.37 (1.85)	0.20	0.55	0.34
ESAS-r-A	50	2.40 (2.17)	9 (18)	49	2.20 (2.34)	8 (16)	-0.25 (2.31)	0.10	0.47	0.08
**Validity measures**										
BSI-18										
Somatization	50	49.24 (9.15)	7 (14)	50	48.26 (8.89)	7 (14)	-0.98 (5.82)	0.2	0.79	0.83
Depression	50	48.32 (8.72)	6 (12)	50	47.06 (8.40)	4 (8)	-1.26 (7.69)	0.16	0.60	0.56
Anxiety	50	47.98 (9.90)	6 (12)	50	45.54 (8.28)	2 (4)	-2.44 (9.89)	0.25	0.41	0.20
GSI	50	47.58 (10.57)	4 (8)	50	45.88 (10.07)	2 (4)	-1.62 (8.77)	0.18	0.65	0.30
PHQ-9	50	3.89 (3.98)	6 (12)	50	3.65 (3.94)	5 (10)	-0.24 (2.99)	0.08	0.72	0.49
GAD-7	50	3.14 (3.39)	3 (6)	50	3.08 (3.29)	3 (6)	-0.06 (2.87)	0.02	0.63	0.29
**Reliability moderators**										
PedsQL										
Physical	50	81.54 (23.65)		50	83.28 (21.50)		1.74 (11.30)	0.15	0.88	
Emotional	49	78.16 (18.84)		50	83.54 (15.57)		5.05 (14.96)	0.34*	0.63	
Social	50	76.65 (24.06)		49	78.57 (24.77)		1.12 (14.83)	0.08	0.81	
School	50	68.50 (20.11)		49	75.92 (19.97)		7.35 (17.53)	0.42**	0.62	
Total	49	76.71 (16.61)		49	80.63 (16.38)		3.92 (10.25)	0.38*	0.81	
LES										
Positive				42	2.31 (3.67)					
Negative				42	-4.88 (5.42)					

^a^ Sample-specific cutoffs for the calculation of Kappas; DT, Distress Thermometer; ESAS-r-D, Edmonton Symptom Assessment-revised-Depression; ESAS-r-A, Edmonton Symptom Assessment-revised-Anxiety; BSI-18, Brief Symptom Inventory-18; GSI, Global Severity Index; PHQ-9, Patient Health Questionnaire-9; GAD-7, Generalized Anxiety Disorder-7; PedsQL, Peds Quality of Life; LES, Life Experiences Survey; *Mdiff* and *SDdiff* are means and standard deviations for T2-T1 differences

* *p* < 0.05

** *p* < 0.01

*** *p* < 0.001.

### Objective 1: Temporal stability

An excellent stability coefficient was found for the DT (ICC = .78, 95% CI .63-.87, r = .79), whereas fair levels were found for the ESAS-r-A (ICC = .47, 95% CI .22-.66, r = .47) and ESAS-r-D (ICC = .55, 95% CI .32-.72, r = .55). When comparing 95% CIs of ICCs, we observed that the DT stability coefficient was larger than the coefficient of the BSI anxiety subscale (ICC = .41, 95% CI .16-.62). No other differences were observed, between the DT and ESAS-r measures or between test measures and validity measures. Only the stability coefficient of the DT was significantly greater that the 0.60 expectation for good-to-excellent stability. There were no mean differences on test measures between test and retest (Table 2). This was also observed with validity measures and confirmed a stable distress level in the sample. With sample-specific cutoffs as with pre-validated cutoffs, Kappas indicated substantial test-retest agreement for the DT, fair agreement for the ESAS-r-D, and slight agreement for the ESAS-r-A [42,43] (Table 2). Stability for non-cases was higher than stability for cases with sample-specific cutoffs (Table 3), but not with pre-validated cutoffs. Interestingly, we did not find significant differences in stability on test measures between mothers and fathers with confidence intervals largely overlapping (ICC values for DT fathers vs mothers = .79 vs .77, ESAS-r-A = .67 vs .36, ESAS-r-D = .63 vs .51). However, these levels suggest a somewhat lower stability of the ESAS-r-A in mothers.

**Table 3:** Stability of Test Measures Using Cutoffs.

			Time 2				
Time 1	Measure (cutoff)^a^		Non case	Case	Total	% Stability	% Difference [CI]
	DT (3+)	Non case	31	1	32	97	
		Case	4	12	16	75	22 [0–44]
		Total	35	13	48	90	
	ESAS-r-D (3+)	Non case	27	4	31	87	
		Case	10	8	18	44	43 [27–59]
		Total	37	12	49	71	
	ESAS-r-A (5+)	Non case	34	6	40	85	
		Case	7	2	9	22	63 [34–92]
		Total	41	8	49	73	

^a^ Sample-specific cutoffs; DT, Distress Thermometer; ESAS-r-D, Edmonton Symptom Assessment-revised-Depression; ESAS-r-A, Edmonton Symptom Assessment-revised-Anxiety; Total percentage stability: (stable Non case + stable Case) / Total N; CI = 95% Confidence Intervals.

To further our understanding of the stability of the DT, we examined stability on the problem list by calculating the % positives at T1 and T2, the proportion of agreement, and the Kappa for all individual problems in the practical, emotional, parenting, family/social, physical, and cognitive domains (S6 Table). We found a median proportion of agreement of .88 (range = .66–1.00) and Kappa of .49 (range = .00–1.00) showing important changes in reported problems over one month. Yet, at the sample level, the most frequent problems at T1 (Fatigue 46%, Feeling tense or nervous 30%, Sleeping difficulties 28%) remained the same over time at T2 (Fatigue 50%, Feeling tense or nervous 36%, Sleeping difficulties 34%).

### Objective 2: Repeatability

**Fig 1:**
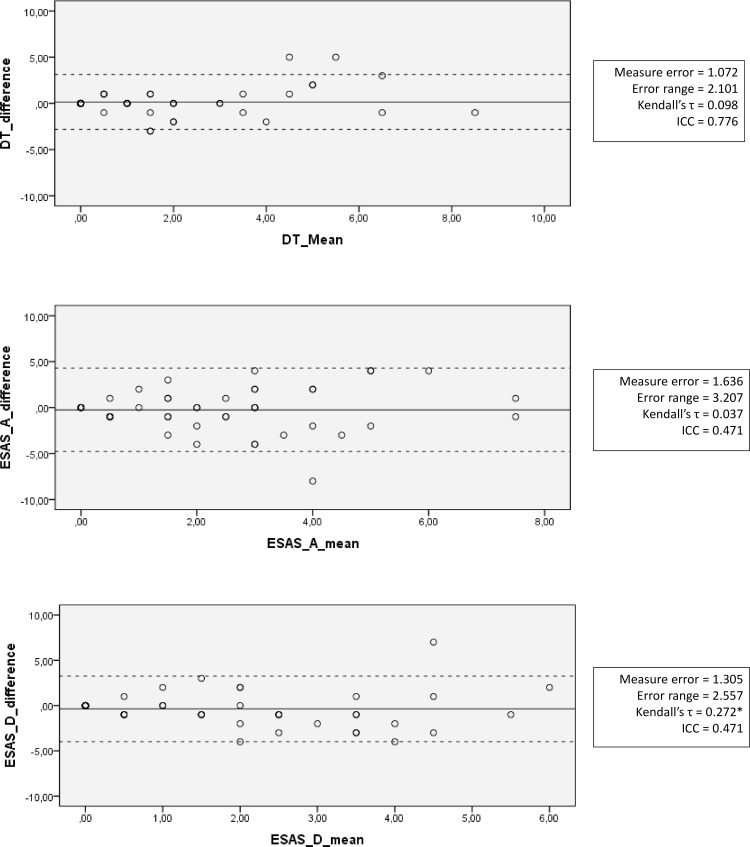
Repeatability analysis of the DT, ESAS-R-A and ESAS-R-D over a 1-month interval. Differences are raw values of T2-T1. Dotted lines indicate the limits of agreement for each measure (M±1.96*SD), in which 95% of differences lie.

From Fig 1, we observe that most of the observed differences on the DT were within 2 points, with the 95% limits of agreement being between -2.82 and +3.12. We also found an almost uniformity in the variance of the repeated measurement (τ = .10, p = .40). The error range indicates a 2.10 above or below the actual measurement. As for the ESAS-r-A, we found a larger bandwidth between limits of agreement of -4.78 and +4.29. Mechanically, the error range on this measure was also larger with 3.21 points below or above actual measurement taken. As on the DT, no relationship of mean with differences was found (τ = .04, p = .74). Regarding the ESAS-r-D we found limits of agreement to be -3.36 and 2.86, and a similar error range as on the DT (2.20 below or above the actual value). For this measure, we found a significant correlation between means and differences suggesting that differences would be larger with higher values of scores (τ = .27, p = .02), suggesting possible higher instability when respondent reported more depression on the first test.

### Objective 3: Moderators of stability on test measures

There was a slight increase in child total QoL between test and retest, particularly on the Emotional and School QoL subscales (Table 2). Instability on test measures was moderately associated with change in child QoL. An increase in child Physical QoL was associated with a decrease in parental distress (DT; *r* = -.39). An increase in child Social QoL was associated with a decrease in parental distress (DT: *r* = -.41), depression (ESAS-r-D: *r* = -.35) and anxiety symptoms (ESAS-r-A: *r* = -.34). An increase in child School QoL was associated with a decrease in parental anxiety (ESAS-r-A: *r* = -.35). There were no association between instability and negative or positive life events. Hierarchical regressions indicated that positive or negative life events did not predict instability in test measures. However, an improvement of the child’s QoL predicted a decrease in parental distress (DT) over time, with an additional 12% of variance explained. This was due to increases in Physical QoL being associated with a decrease in DT over time. Stability levels for the ESAS-r items were unrelated to life events or child QoL (Table 4).

**Table 4:** Summary of Hierarchical Regressions Predicting Instability of Test Measures.

	**Time 2 (retest)**					
	**DT**		**ESAS-r-D**		**ESAS-r-A**	
**Predictor**	Δ*R*^*2*^	**β**	Δ*R*^*2*^	**β**	Δ*R*^*2*^	**β**
Block 1	0.58***		0.22**		0.15*	
Time 1 (test)		0.76***		0.47**		0.38*
Block 2a	0.01		0.07		0.08	
Negative life events		0.11		0.23		0.26
Positive life events		-0.02		-0.17		-0.18
Block 2b	0.12*		0.14		0.15	
Physical QoL change		-0.23*		-0.17		0.01
Emotional QoL change		0.08		-0.11		0.02
Social QoL change		-0.17		-0.22		-0.25
School QoL change		-0.11		0.02		-0.24

DT, Distress Thermometer; ESAS-r-D, Edmonton Symptom Assessment-revised-Depression; ESAS-r-A, Edmonton Symptom Assessment-revised-Anxiety; Change is defined as T2-T1 for child QoL

* *p* < 0.05

** *p* < 0.01

*** *p* < 0.001.

The full database used for this study is available (S7 Table).

## Discussion

This study was the first to investigate stability and repeatability in the DT and the Depression and Anxiety items of the ESAS-r with parents of paediatric cancer survivors. As hypothesized at a one-month interval, we found excellent stability for the DT and only fair stability for the ESAS-r-D and ESAS-r-A. Repeatability analyses showed large error ranges for the three tools and a lower accuracy of measurement for the ESAS-r-A than the DT or even the ESAS-r-D. Changes in children's QoL over time, particularly on the physical component, predicted instability on the DT.

These levels of stability may be due to a variety of factors. Consistent with our study design, there was negligible change in distress over time as measured by test and validity measures. As the measured construct appears stable over time, explanations for instability most probably lie with measurement. High levels of stability could be interpreted as high reliability whereas low levels of stability could not be positively interpreted as a lack of reliability. Parents with different levels of distress also tended to remain at the same level over time on the DT, but less so on the ESAS-r items. Parents who did not report clinical levels of distress were more likely to stay in that category, as opposed to parents who first reported clinical levels of distress. This may be accounted for by the lower base rate for cases, but it could also describe that parental distress is experimented as a normative transitory phenomenon at this stage of the cancer trajectory.

Taking into account the impact of time on stability, it is coherent that the DT test-retest coefficient with caregivers at a month interval was similar to the one reported with adult patients in remission at a week interval [19]. This indicates good temporal stability for the DT. Unexpectedly, test-retest coefficients for the ESAS-r items in this study were larger than those reported with adult patients at a week interval [21]. This may result from changes in patients' distress over time in the latter study, which mechanically decreased the stability coefficient.

The different levels of stability of the DT and ESAS-r items are probably due to their time frames [24]. On the DT, participants evaluate an average week level of distress, whereas they report on a day level on the ESAS-r. As a consequence, higher sensitivity or lower stability is expected on the ESAS-r. The ESAS was originally designed to be used twice a day [12] and most studies investigating its stability have selected hours to days intervals [21–23, 44–46]. This stresses the fact that lower stability over longer periods of time for such an instrument is a sought-after property as it may reflect sensitivity. Yet, with the present design, it was not possible to disentangle measurement error from change in experienced anxiety and depression. The DT's larger stability coefficient might also be partially explained by the overarching term of *distress*, which allows participants to include various manifestations of emotional difficulties, as opposed to specific symptoms on the ESAS-r. We expect broader categories to be more stable than specific transient symptoms. Although an examination of sensitivity is beyond the scope of the current study as we focused on a stable situation instead of a changing situation, we observed that even slight changes on test measures over time were closely associated with slight changes on validity measures with the range of associations suggesting that sensitivity varies across these instruments (DT with GSI: r = .46, ESAS-r-A with GAD-7: r = .64, ESAS-r-D with PHQ-9: r = .75). A full sensitivity study should be led in the future in situations were distress is expected to vary.

When comparing our findings to other test-retest coefficients, it is important to keep in mind that test-retest coefficients for emotional experience are not likely to be as strong as those of more enduring personality traits, such as Extraversion (stability over two months: .89 [47]), since the proportion of expected true change is greater for the former [24]. Moreover, although larger tests usually show stronger stability than shorter tests because they are less vulnerable to chance or settings elements [48], the one-item DT test-retest coefficient was larger than the coefficient of the multi-item BSI anxiety subscale. The DT appeared as a stable instrument with parents of survivors, beyond a longer screener, which speaks in favour of its reliability.

Our findings also confirm that this tool has good convergent validity. We found different cutoffs than in the previous literature [13, 16], which may be indicative of the different levels of distress. For example, parents of survivors tend to report lower levels of distress than parents of children in treatment. Screening for distress requires using cutoffs that are suited for each population. Therefore, although the use of the DT with caregivers is still rare, our data support future research on its use with this population in paediatric oncology [9]. As for the ESAS-r items, this study is not able to disentangle a desired sensitivity from a lack of stability of the scales. Future research should use appropriate time-lapses (i.e., one day). Given their lower stability, the ESAS-r-D and -A should probably be used in situations where day-to-day changes are expected. In contrast, the DT would be more appropriate in contexts where changes are expected on a longer period (i.e. weeks or months).

When focusing on accuracy of measurement, repeatability analyses revealed that the error bandwidth was large for such 11-points instruments, especially for the ESAS-r-A. In that case, a self-reported level of 5 for instance, should be considered as a figure comprised between 1.79 and 8.21. This bandwidth covers more than 64% of the scale maximum range, which is a large ‘error.’ Even in the case of the DT, the ‘error’ covers 42% of the scale. As for stability, these repeatability indices should be analyzed in the context of lapse between time-points and time frame of instruments.

When exploring factors associated with stability, we found that variations in parental distress were related to changes in children’s QoL. Although children were clinically stable, their emotional and school QoL increased over time. With test assessments being taken over the summer and retests once school had resumed, it is possible that a more structured routine contributed to improvements on these domains of children's QoL. Changes in children's physical, social, and school QoL between test and retest were moderately associated with instability in test measures, implying that an increase in child QoL was associated with a decrease in parental distress. Although expected, this association suggests that the test measures could be sensitive to small changes in the parent's environment and that attention should be given to families when the child's status changes. Children's physical health appeared to have a high level of impact on parents’ well-being over time, consistent with a previous report [49]. It is possible that a more observable ability (physical QoL) stands as a stronger factor of distress change as opposed to psychological QoL. Parents of cancer survivors may also be more attuned to subtle physical changes in their child's health, considering their significant and durable involvement during treatment. Future studies could examine other moderators of stability such as gender and investigate the impact of different time-lapses between measures.

### Limitations

The present sample size is relatively small for stability research, thus yielding less accurate stability estimates because of larger confidence intervals [24]. However, it is typical of clinical reports, especially in paediatric oncology where numbers are low. In addition, our focus on parents during the survivorship period probably limits the applicability of the results to other situations. Yet, this made it possible to study the stability of measures in a clinically-stable situation where distress was also relatively stable. The fact that the sample included 17 couples with both mother and father participating to the study violates assumptions of independence of observations. To deal with this issue, we performed additional analyses comparing stability in 10 randomly selected independent respondents samples (N = 33) from the database and found results supporting our interpretations (Min-Max ICCs: DT = .71-.77, ESAS-r-A = .38-.50, ESAS-r-D = .40-.55). Considering that 16% of parents had difficulty understanding the LES instructions, the reported absence of association should not be taken as an absence of impact of life events in future studies. Alternate instructions or another instrument could be used. Finally, the QoL proxy measure may reflect how parents perceive their child’s QoL instead of the child’s actual QoL. This perception could well be influenced by parental distress. Therefore, it is possible that changes in distress influenced responses on the QoL inventory, a phenomenon that our design cannot identify. Despite these limitations, the assets of the study relate to the inclusion of a population with a stable status, a theoretically-based time interval and expected associations, as well as comparisons with reference measures. Because families are followed less frequently after treatment and parents’ involvement decreases gradually during the transition period, it is all the more important to make use of contacts with parents to screen for distress in the family.

## Conclusions

We conducted a detailed examination of the temporal stability and repeatability of the DT, ESAS-r-D and ESAS-r-A with parents of children who have had cancer. We found that the DT showed excellent stability over time, and the ESAS-r items had fair stability levels. The DT was not significantly more stable than the ESAS-r items though. Error ranges were large for the three test measures. Although high stability spoke in favor of reliability if the DT in a stable situation, it was not possible to interpret low stability in ESAS-r measures because of a shorter time-frame on this instrument. Future studies should address this limitation by investigating different time-lapses and sensitivity in situations where change in distress is expected or provoked. We found that stability levels on the DT were associated with changes in children’s status as reported by parents. No impact of life events was reported. Future studies could also examine other moderators of stability such as gender. Parental distress screening in follow-up care should rely on psychometrically sound instruments, both reliable and sensitive, which would be able to assist health teams in further helping parents in need.

## Supporting Information

S1 File Blank copy of questionnaire. (DOCX)

S1 Table Diagnostic Accuracy of the DT against GSI (BSI-18; *n* = 50). (DOCX)

S2 Table Diagnostic Accuracy of the DT against PHQ-9 (*n* = 50). (DOCX)

S3 Table Diagnostic Accuracy of the DT against GAD-7 (*n* = 50). (DOCX)

S4 Table Diagnostic Accuracy of the ESAS-r-D against PHQ-9 (*n* = 50). (DOCX)

S5 Table Diagnostic accuracy of the ESAS-r-A against GAD-7 (*n* = 50). (DOCX)

S6 Table Repeatability for 39 problems from the problem list of the Distress Thermometer administered to 50 parents of young survivors of child cancer at a 1-month interval (n = 50). (DOCX)

S7 Table Database. (XLSX)
