# Supplementary material for: Stability and Repeatability of the Distress Thermometer (DT) and the Edmonton Symptom Assessment System-Revised (ESAS-r) with Parents of Childhood Cancer Survivors
Source: PLoS One. 2016 Jul 25;11(7):e0159773. doi: 10.1371/journal.pone.0159773 (PMC4959708; doi:10.1371/journal.pone.0159773)
Supplement: S1 File — (DOCX) [file pone.0159773.s001.docx]

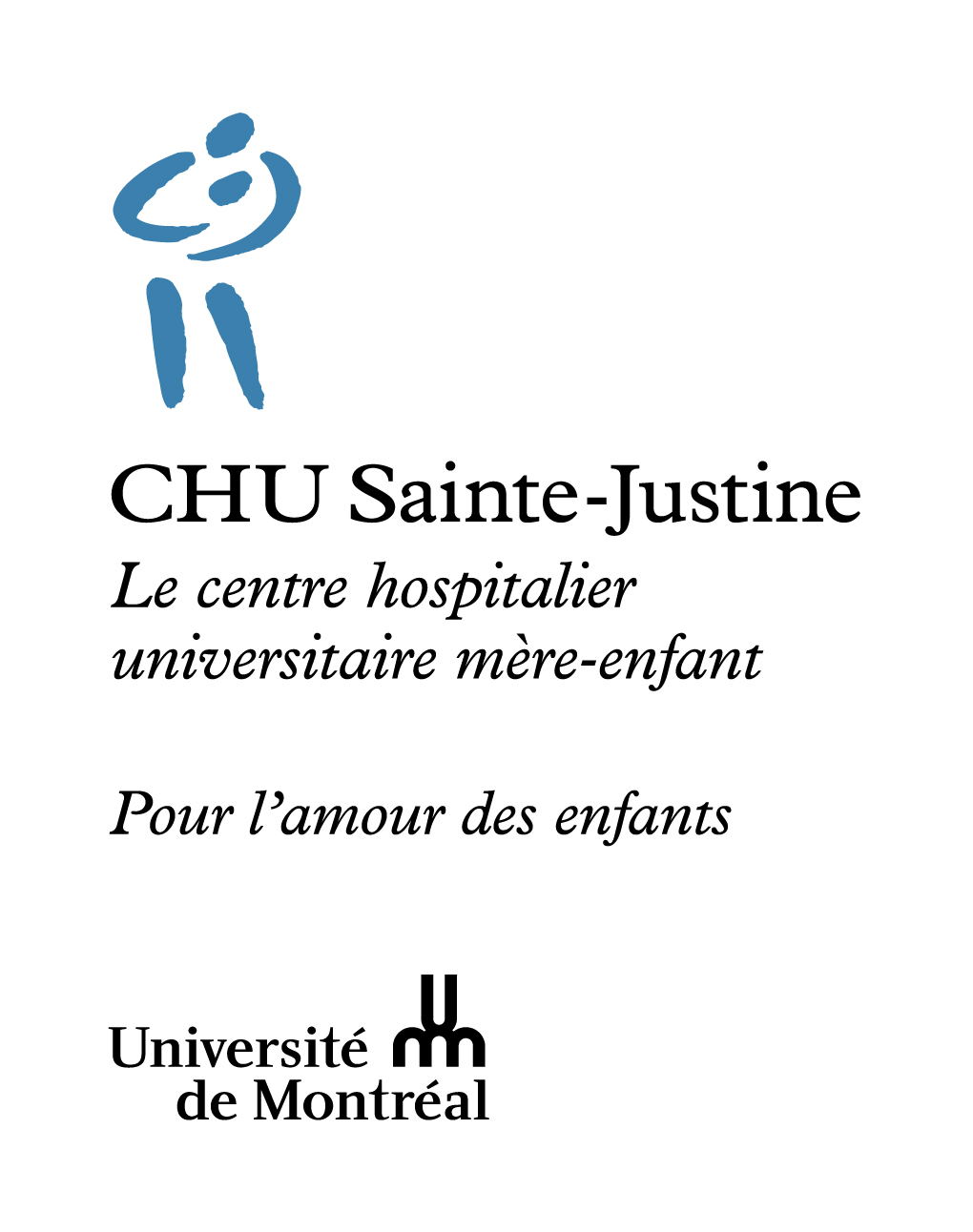


**S1 File. Blank copy of questionnaire**

**RESEARCH PROJECT**

Étude DETECT-parents

**Distress thermometer-Parents** (Haverman et al., 2013)

**Edmonton Symptom Assessment System-R (-A et -D)** (Watanabe et al., 2011)

*Distress: unpleasant emotion that diminishes one’s quality of life and can be detrimental to one’s functioning.*

**Edmonton Symptom Assessment System-Revised**Please circle the number that best describes how you feel **NOW**

| **No depression** | 0 1 2 3 4 5 6 7 8 9 10 | **Worst possible depression** |
| --- | --- | --- |
| *(depression = feeling sad)* |  |  |
| **No anxiety** | 0 1 2 3 4 5 6 7 8 9 10 | **Worst possible anxiety** |
| *(anxiety = feeling nervous)* |  |  |

**Problem list (parents):** Check if any of the following applies/has applied to you in the **PAST WEEK (including today)**

| **Practical problems** |  | **Family/social problems** |
| --- | --- | --- |
| ❑ Housing |  | ❑ Dealing with (ex)partner |
| ❑ Work/study |  | ❑ Dealing with family |
| ❑ Finances/insurance |  | ❑ Dealing with friends |
| ❑ Housekeeping |  | ❑ Interacting with your child(ren) |
| ❑ Transport |  |  |
| ❑ Child care/child supervision |  |  |
| ❑ Leisure activities/relaxing |  |  |

| **Emotional problems** |  | **Physical problems** |
| --- | --- | --- |
| ❑ Keeping emotions under control |  | ❑ Eating |
| ❑ Self-confidence |  | ❑ Weight |
| ❑ Fears |  | ❑ Sleep |
| ❑ Depression |  | ❑ Fatigue |
| ❑ Feeling tense or nervous |  | ❑ Out of shape/condition |
| ❑ Loneliness |  | ❑ Pain |
| ❑ Feelings of guilt |  | ❑ Sexuality |
| ❑ Use of substances (alcohol, drugs, medication) |  |  |
| ❑ Intrusive/recurrent thoughts about a specific event |  |  |

| **Parenting your child diagnosed with cancer** |  | **Cognitive problems** |
| --- | --- | --- |
| ❑ Dealing with your child |  | ❑ Concentration |
| ❑ Dealing with the feelings of your child |  | ❑ Memory |
| ❑ Talking about the disease/consequences with your   child |  |  |
| ❑ Independence of your child |  |  |
| ❑ Following advice about treatment/Giving your   child his/her medication |  |  |

| **Additional questions** |
| --- |
| ❑ I feel that I have received enough support from people around me.   If yes, what kind of support (practical, emotional, etc.)? |
| ❑ People often react to my situation with a lack of understanding. |
| ❑ I have a chronic disease. |
| ❑ I get along very well/well/ fair/ poor with the medical staff. |
| ❑ I would like to talk to a professional about my situation. |

**Distress Thermometer**

Check how much distress you have been experiencing in the **PAST WEEK**

**(including today)**

**Brief Symptom Inventory-18**(BSI-18; Derogatis, 2001)

Please read each sentence carefully, and circle the number that best describes **how much that problem has distressed or bothered you during the PAST 7 DAYS INCLUDING TODAY.**

| **HOW MUCH WERE YOU DISTRESSED BY** | **Not at all** | **A little bit** | **Moderately** | **Quite a bit** | **Extremely** |
| --- | --- | --- | --- | --- | --- |
| 1. Faintness or dizziness | 0 | 1 | 2 | 3 | 4 |
| 1. Feeling no interest in things | 0 | 1 | 2 | 3 | 4 |
| 1. Nervousness or shakiness inside | 0 | 1 | 2 | 3 | 4 |
| 1. Pains in heart or chest | 0 | 1 | 2 | 3 | 4 |
| 1. Feeling lonely | 0 | 1 | 2 | 3 | 4 |
| 1. Feeling tense or keyed up | 0 | 1 | 2 | 3 | 4 |
| 1. Nausea or upset stomach | 0 | 1 | 2 | 3 | 4 |
| 1. Feeling blue | 0 | 1 | 2 | 3 | 4 |
| 1. Suddenly scared for no reason | 0 | 1 | 2 | 3 | 4 |
| 1. Trouble getting your breath | 0 | 1 | 2 | 3 | 4 |
| 1. Feeling of worthlessness | 0 | 1 | 2 | 3 | 4 |
| 1. Spells of terror or panic | 0 | 1 | 2 | 3 | 4 |
| 1. Numbness or tigling in parts of your body | 0 | 1 | 2 | 3 | 4 |
| 1. Feeling hopeless about the future | 0 | 1 | 2 | 3 | 4 |
| 1. Feeling so restless you couldn’t sit still | 0 | 1 | 2 | 3 | 4 |
| 1. Feeling weak in parts of your body | 0 | 1 | 2 | 3 | 4 |
| 1. Thoughs of ending your life | 0 | 1 | 2 | 3 | 4 |
| 1. Feeling fearful | 0 | 1 | 2 | 3 | 4 |

**General Anxiety Disorder-7**

(GAD-7; Spitzer et al., 2006)

| **Oven the LAST TWO WEEKS, how often have you been bothered by the following problems?**  (Use « 🗸» to indicate your answer) | **Not at all** | **Several days** | **More than half the days** | **Nearly every day** |
| --- | --- | --- | --- | --- |
| 1. Feeling nervous, anxious or on edge | 0 | 1 | 2 | 3 |
| 2. Not being able to stop or control worrying | 0 | 1 | 2 | 3 |
| 3. Worrying too much about different things | 0 | 1 | 2 | 3 |
| 4. Trouble relaxing | 0 | 1 | 2 | 3 |
| 5. Being so restless that it is hard to sit still | 0 | 1 | 2 | 3 |
| 6. Becoming easily annoyed or irritable | 0 | 1 | 2 | 3 |
| 7. Feeling afraid as if something awful  might happen | 0 | 1 | 2 | 3 |

**Patient Health Questionnaire-9**

(PHQ-9; Kroenke et al., 2001)

| **Oven the LAST TWO WEEKS, how often have you been bothered by any of the following problems?**  (Use « 🗸» to indicate your answer) | **Not at all** | **Several days** | **More than half the days** | **Nearly every day** |
| --- | --- | --- | --- | --- |
| 1. Little interest or pleasure in doing things | 0 | 1 | 2 | 3 |
| 2. Feeling down, depressed, or hopeless | 0 | 1 | 2 | 3 |
| 3. Trouble falling or staying asleep, or sleeping too  much | 0 | 1 | 2 | 3 |
| 4. Feeling tired or having little energy | 0 | 1 | 2 | 3 |
| 5. Poor appetite or overeating | 0 | 1 | 2 | 3 |
| 6. Feeling bad about yourself — or that you are a  failure or have let yourself or your family down | 0 | 1 | 2 | 3 |
| 7. Trouble concentrating on things, such as   reading the newspaper or watching television | 0 | 1 | 2 | 3 |
| 8. Moving or speaking so slowly that other people  could have noticed? Or the opposite — being   so fidgety or restless that you have been   moving around a lot more than usual | 0 | 1 | 2 | 3 |
| 9. Thoughts that you would be better off dead or of  hurting yourself in some way | 0 | 1 | 2 | 3 |

If you checked off any problems, how difficult have these problems made it for you to do your work, take care of things at home, or get along with other people?

Not difficult Somewhat Very Extremely

at all difficult difficult difficult

☐ ☐ ☐ ☐

**Pediatric Quality of Life Inventory 4.0, Generic Score Scales,
Parent report for young children (age 5-7)**

(PedsQL; Varni, Seid, & Rode, 1999)

On the following page is a list of this that might be a problem for **your child**. Please tell us **how much of a problem** each one has been for **your child** during the **PAST MONTH** by circling: 0 if it is never a problem, 1 if it is almost never a problem, 2 if it is sometimes a problem, 3 if it is often a problem, 4 if it is almost always a problem. There is no right or wrong answers. If you do not understand a question, please ask for help.

| **PHYSICAL FUNCTIONING (problems with…)** | **Never** | **Almost never** | **Some-times** | **Often** | **Almost always** |
| --- | --- | --- | --- | --- | --- |
| 1. Walking more than one block | 0 | 1 | 2 | 3 | 4 |
| 2. Running | 0 | 1 | 2 | 3 | 4 |
| 3. Participating in sports activities or exercise | 0 | 1 | 2 | 3 | 4 |
| 4. Lifting something heavy | 0 | 1 | 2 | 3 | 4 |
| 5. Taking a bath or shower by himself or herself | 0 | 1 | 2 | 3 | 4 |
| 6. Doing chores, like picking up his or her toys | 0 | 1 | 2 | 3 | 4 |
| 7. Having hurts or aches | 0 | 1 | 2 | 3 | 4 |
| 8. Low energy level | 0 | 1 | 2 | 3 | 4 |
| **EMOTIONAL FUNCTIONING (problems with…)** | **Never** | **Almost never** | **Some-times** | **Often** | **Almost always** |
| 1. Feeling afraid or scared | 0 | 1 | 2 | 3 | 4 |
| 2. Feeling sad or blue | 0 | 1 | 2 | 3 | 4 |
| 3. Feeling angry | 0 | 1 | 2 | 3 | 4 |
| 4. Trouble sleeping | 0 | 1 | 2 | 3 | 4 |
| 5. Worrying about what will happen to him or her | 0 | 1 | 2 | 3 | 4 |
| **SOCIAL FUNCTIONING (problems with…)** | **Never** | **Almost never** | **Some-times** | **Often** | **Almost always** |
| 1. Getting along with other children | 0 | 1 | 2 | 3 | 4 |
| 2. Other children not wanting to be his or her friend | 0 | 1 | 2 | 3 | 4 |
| 3. Getting teased by other children | 0 | 1 | 2 | 3 | 4 |
| 4. Not able to do things that other children his or her age  can do | 0 | 1 | 2 | 3 | 4 |
| 5. Keeping up when playing with other children | 0 | 1 | 2 | 3 | 4 |
| **SCHOOL FUNCTIONING (problems with…)** | **Never** | **Almost never** | **Some-times** | **Often** | **Almost always** |
| 1. Paying attention in class | 0 | 1 | 2 | 3 | 4 |
| 2. Forgetting things | 0 | 1 | 2 | 3 | 4 |
| 3. Keeping up with school activities | 0 | 1 | 2 | 3 | 4 |
| 4. Missing school because of not feeling well | 0 | 1 | 2 | 3 | 4 |
| 5. Missing school to go to the doctor or hospital | 0 | 1 | 2 | 3 | 4 |

According to you, since the last month, your child’s health status has gotten: ❑ better ❑ worse ❑ is stable

**Socio-demographic questionnaire and clinical history**

1. **INFORMATION ABOUT THE PARENT**

**Who are you?**

❑ Mother ❑ Father ❑ Other ____________________________
 **Date and place of birth** (mm/dd/yyyy) _______________________________

**Country of origin** ____________________________

**Number of years in Canada** ____________________________

**Civil status**

❑ single ❑ divorced ❑ separated ❑ widow

❑ married ❑ common law **Education**

❑ High-school ❑ CEGEP ❑ University

**Occupation/studies (description/title)** ____________________________

**Education/work status?**

❑ Full time ❑ Part time ❑ Not working ❑ Retired

**Annual income**

❑ < 20 000$ ❑ 20 000-40 000$ ❑ 40 000-60 000$ ❑ 60 000-80 000$ ❑ > 80 000$

1. **INFORMATION ABOUT THE FAMILY**

**Date and place of birth of your child** (mm/dd/yyyy) _________________________________

**Family structure of your child** (check all that applies)

❑ Father alive

❑ Mother alive

❑ Sister(s) Number____

❑ Brother(s) Number____

❑ Other(s) All other member in the immediate family ____________________________

**Do you live with your child** (main residence)? ❑ yes ❑ no

**Do you also live with** (check all that applies)

❑ Father/mother of the child
❑ Brothers/sisters of the child
❑ Other (roommates/family)

**Involvement of the other parent in the child’s medical care**

❑ yes ❑ no

**Distance from your home to the treatment center** ❑ < 10 km ❑ 11-50 km ❑ > 50 km

1. **TREATMENT/DISEASE HISTORY OF YOUR CHILD**

**Diagnosis date** ____________________________

**Type of diagnosis**

❑ Brain tumour Specify ____________________________

❑ Other solid tumour Specify ____________________________

**Relapse** ❑ no ❑ yes Specify when ____________________________

**Recent infections** (past three months) ❑ no ❑ yes Specify ____________________________
 **Principal cancer treatments received**

❑ Surgery

❑ Chemotherapy

❑ Radiation therapy

**Treatment changes**

❑ yes ❑ no

Specify why_______________________________________________________________________

Specify date of change____________________________

**Type of follow-up treatment** _________________________________________________________

1. **PSYCHOLOGICAL HEALTH OF THE PARENT**

**Antecedents**

Before your child’s cancer diagnosis, did you experience stressful life events (e.g., divorce, moving) or did you suffer from psychological problems (e.g., depression, anxiety symptoms, etc. or psychological diagnosis)?
❑ yes ❑ no

If yes, please specify ________________________________________________________________ _________________________________________________________________________________

**Psychological difficulties**

Did you ever consult someone for psychological issues in association with your child’s cancer (social worker, psychologist, psychiatrist, art therapist, etc.) ?

❑ yes ❑ no

If yes, please specify

who ? ________________________________________________________

when ? ___________________________

Did you receive a treatment? ❑ yes ❑ no
If yes, please specify the type and duration

________________________________________________________________________
