# Supplementary material for: Stability and Repeatability of the Distress Thermometer (DT) and the Edmonton Symptom Assessment System-Revised (ESAS-r) with Parents of Childhood Cancer Survivors
Source: PLoS One. 2016 Jul 25;11(7):e0159773. doi: 10.1371/journal.pone.0159773 (PMC4959708; doi:10.1371/journal.pone.0159773)
Supplement: S2 Table — (DOCX) [file pone.0159773.s003.docx]

**Table S2. Diagnostic Accuracy of the DT against PHQ-9 (*n* = 50).**

|  |  |  | **PHQ-9 (AUC = 0.88)** | | | |
| --- | --- | --- | --- | --- | --- | --- |
| **DT score** | **n** | **% cum** | **Sen** | **Spe** | **PPV** | **NPV** |
| 0 | 19 | 38 | 1.00 | 0.00 | 0.12 | 0.00 |
| 1 | 10 | 58 | 1.00 | 0.43 | 0.19 | 1.00 |
| 2 | 5 | 68 | 1.00 | 0.66 | 0.29 | 1.00 |
| 3 | 8 | 84 | 0.83 | 0.75 | 0.31 | 0.97 |
| 4 | 4 | 92 | 0.50 | 0.89 | 0.38 | 0.93 |
| 5 | 2 | 96 | 0.33 | 0.96 | 0.50 | 0.91 |
| 6 | 0 | - | - | - | - | - |
| 7 | 1 | 98 | 0.33 | 1.00 | 1.00 | 0.92 |
| 8 | 0 | - | - | - | - | - |
| 9 | 1 | 100 | 0.17 | 1.00 | 1.00 | 0.90 |
| 10 | 0 | - | - | - | - | - |

AUC, Area Under the Curve; % cum, % cumulative; Sen,
Sensitivity; Spe, Specificity; PPV, Positive Predictive Value; NPV,
Negative Predictive Value.
