# Supplementary material for: Stability and Repeatability of the Distress Thermometer (DT) and the Edmonton Symptom Assessment System-Revised (ESAS-r) with Parents of Childhood Cancer Survivors
Source: PLoS One. 2016 Jul 25;11(7):e0159773. doi: 10.1371/journal.pone.0159773 (PMC4959708; doi:10.1371/journal.pone.0159773)
Supplement: S4 Table — (DOCX) [file pone.0159773.s005.docx]

**Table S4. Diagnostic Accuracy of the ESAS-r-D against PHQ-9 (*n* = 50).**

|  |  |  | **PHQ-9 (AUC = 0.91)** | | | |
| --- | --- | --- | --- | --- | --- | --- |
| **ESAS-r-D score** | **n** | **% cum** | **Sen** | **Spe** | **PPV** | **NPV** |
| 0 | 18 | 36 | 1.00 | 0.00 | 0.12 | 0.00 |
| 1 | 10 | 54 | 1.00 | 0.41 | 0.19 | 1.00 |
| 2 | 4 | 64 | 1.00 | 0.64 | 0.27 | 1.00 |
| 3 | 5 | 74 | 1.00 | 0.73 | 0.33 | 1.00 |
| 4 | 6 | 86 | 0.83 | 0.82 | 0.38 | 0.97 |
| 5 | 4 | 94 | 0.67 | 0.93 | 0.57 | 0.95 |
| 6 | 3 | 100 | 0.17 | 0.96 | 0.33 | 0.89 |
| 7 | 0 | - | - | - | - | - |
| 8 | 0 | - | - | - | - | - |
| 9 | 0 | - | - | - | - | - |
| 10 | 0 | - | - | - | - | - |

AUC, Area Under the Curve; % cum, % cumulative; Sen, Sensitivity; Spe,
Specificity; PPV, Positive Predictive Value; NPV, Negative Predictive
Value.
