# Supplementary material for: Stability and Repeatability of the Distress Thermometer (DT) and the Edmonton Symptom Assessment System-Revised (ESAS-r) with Parents of Childhood Cancer Survivors
Source: PLoS One. 2016 Jul 25;11(7):e0159773. doi: 10.1371/journal.pone.0159773 (PMC4959708; doi:10.1371/journal.pone.0159773)
Supplement: S5 Table — (DOCX) [file pone.0159773.s006.docx]

**Table S5. Diagnostic accuracy of the ESAS-r-A against GAD-7 (*n* = 50).**

|  |  |  | **GAD-7 (AUC = 0.96)** | | | |
| --- | --- | --- | --- | --- | --- | --- |
| **ESAS-r-A score** | **n** | **% cum** | **Sen** | **Spe** | **PPV** | **NPV** |
| 0 | 11 | 22 | 1.00 | 0.00 | 0.06 | 0.00 |
| 1 | 10 | 42 | 1.00 | 0.23 | 0.08 | 1.00 |
| 2 | 7 | 56 | 1.00 | 0.45 | 0.10 | 1.00 |
| 3 | 11 | 78 | 1.00 | 0.60 | 0.14 | 1.00 |
| 4 | 2 | 82 | 1.00 | 0.83 | 0.27 | 1.00 |
| 5 | 4 | 90 | 1.00 | 0.87 | 0.33 | 1.00 |
| 6 | 2 | 94 | 0.67 | 0.94 | 0.40 | 0.98 |
| 7 | 1 | 96 | 0.67 | 0.98 | 0.67 | 0.98 |
| 8 | 2 | 100 | 0.33 | 0.98 | 0.50 | 0.96 |
| 9 | 0 | - | - | - | - | - |
| 10 | 0 | - | - | - | - | - |

AUC, Area Under the Curve; % cum, % cumulative; Sen, Sensitivity; Spe,
Specificity; PPV, Positive Predictive Value; NPV, Negative Predictive
Value.
