# Supplementary material for: Stability and Repeatability of the Distress Thermometer (DT) and the Edmonton Symptom Assessment System-Revised (ESAS-r) with Parents of Childhood Cancer Survivors
Source: PLoS One. 2016 Jul 25;11(7):e0159773. doi: 10.1371/journal.pone.0159773 (PMC4959708; doi:10.1371/journal.pone.0159773)
Supplement: S6 Table — (DOCX) [file pone.0159773.s007.docx]

| **Table S6. Repeatability for 39 problems from the problem list of the DT administered to 50 parents of young survivors of child cancer at a 1-month interval** | | | | |
| --- | --- | --- | --- | --- |
| DT problems* | % positive response T1 | % positive response T2 | Proportion in agreement | Kappa |
| Practical 1 | 0 | 4 | 96 | N/A |
| Practical 2 | 28 | 22 | 70 | 0.204 |
| Practical 3 | 20 | 16 | 92 | 0.730 |
| Practical 4 | 26 | 16 | 82 | 0.466 |
| Practical 5 | 2 | 4 | 98 | 0.658 |
| Practical 6 | 16 | 18 | 86 | 0.504 |
| Practical 7 | 14 | 22 | 84 | 0.464 |
| Emotional 1 | 22 | 22 | 80 | 0.417 |
| Emotional 2 | 12 | 12 | 88 | 0.432 |
| Emotional 3 | 10 | 10 | 84 | 0.111 |
| Emotional 4 | 6 | 8 | 94 | 0.540 |
| Emotional 5 | 30 | 36 | 66 | 0.234 |
| Emotional 6 | 6 | 6 | 94 | 0.645 |
| Emotional 7 | 16 | 8 | 88 | 0.440 |
| Emotional 8 | 2 | 2 | 100 | 1.000 |
| Emotional 9 | 4 | 0 | 96 | N/A |
| Parenting 1 | 2 | 2 | 96 | -0.020 |
| Parenting 2 | 12 | 4 | 88 | 0.202 |
| Parenting 3 | 10 | 10 | 92 | 0.556 |
| Parenting 4 | 8 | 10 | 86 | 0.146 |
| Parenting 5 | 8 | 2 | 94 | 0.380 |
| Family/Social 1 | 20 | 22 | 74 | 0.217 |
| Family/Social 2 | 6 | 2 | 92 | -0.031 |
| Family/Social 3 | 2 | 6 | 96 | 0.485 |
| Family/Social 4 | 20 | 14 | 90 | 0.648 |
| Physical 1 | 6 | 6 | 92 | 0.291 |
| Physical 2 | 18 | 16 | 86 | 0.504 |
| Physical 3 | 28 | 34 | 78 | 0.488 |
| Physical 4 | 46 | 50 | 84 | 0.680 |
| Physical 5 | 22 | 18 | 84 | 0.501 |
| Physical 6 | 20 | 14 | 86 | 0.507 |
| Physical 7 | 8 | 8 | 96 | 0.728 |
| Cognitive 1 | 26 | 18 | 88 | 0.654 |
| Cognitive 2 | 10 | 12 | 94 | 0.694 |
| Additional 1 | 38 | 24 | 66 | 0.223 |
| Additional 2 | 8 | 10 | 86 | 0.146 |
| Additional 3 | 2 | 4 | 98 | 0.658 |
| Additional 4 | 40 | 30 | 78 | 0.522 |
| Additional 5 | 0 | 0 | 100 | N/A |

Note. * Problem list from: Haverman L, van Oers HA, Limperg PF, Houtzager BA, Huisman J, Darlington AS, et al. Development and validation of the distress thermometer for parents of a chronically ill child. J Pediatr. 2013;163(4)
